# Role of cardiac imaging in Anderson-Fabry cardiomyopathy

**DOI:** 10.1186/s12947-019-0151-5

**Published:** 2019-01-23

**Authors:** Walter Serra, Nicola Marziliano

**Affiliations:** 1 Cardiology Division, Surgery Department, University Hospital-Parma, Via Antonio Gramsci 14, 43100 Parma, IT Italy; 2 University of Molise, Health Sciences Department-Campobasso, Campobasso, IT Italy; 3 Fondazione Floresta Longo, Catania, IT Italy

## Abstract

The Anderson-Fabry disease (AFD, or simply Fabry Disease, FD; MIM #301500) is a rare X-linked lysosomal storage disorder (Xq22.1) characterized by progressive renal failure, leading to morbidity through cardio- and cerebro-vascular involvement. Despite the classic phenotype, only cardiac involvement (cardiac variant of AFD; MIM 301500) is frequent in about 40% of male and 28% of female AFD patients, as reported by the Fabry Registry (https://www.registrynxt.com). Morphologically, the cardiac characteristic of the disease, occurs as left ventricular hypertrophy, is accompanied by myocardial fibrosis. Cardiologists may come across these patients during clinical and instrumental evaluation in individuals with non-specific symptoms such as chest pain and arrhythmias, or after instrumental evidence of left ventricular hypertrophy/hypertrophic cardiomyopathy (HCM; MIM 192600). A comprehensive cardiological work-up, including a cardiological visit, a baseline electrocardiogram (ECG) and imaging by both echocardiography (ECHO) and magnetic resonance (MRI) enables identification of the cardiac involvement in patients with a proven diagnosis of AFD. The heart involvement is present in up to 75% of AFD patients irrespective of their sex. Involvement includes ECG and echocardiography features which suggest AFD and not HCM. Cardiac imaging plays an important role in detecting this sub-type of cardiomyopathy, which, since 2001, has benefited from the introduction of the enzyme replacement therapy (ERT) in symptomatic and pre-symptomatic patients.

## Introduction

Many storage diseases are associated with left ventricular cardiac hypertrophy (LVCH; Table 1). Anderson-Fabry disease (AFD) is the most common form of X-linked lysosomal disorder (Xq22.1), with cardiac involvement having a prevalence of 0.5–1% in patients with hypertrophic cardiomyopathy (HCM; [1, 2]) typically at the beginning of adulthood. In the classic AFD form (MIM # 301500), over 60% of patients of both sexes show cardiovascular signs such as arrhythmias, valve abnormalities and cardiomyopathy. Affected males develop cardiac features around the second decade of life: LVCH, valve diseases and conduction alterations are the earliest signs. Cardiac involvement tends to increase with age, both in incidence and severity, and represents, along with untreated renal failure, the first cause of death among patients with AFD [3, 4].

**Table 1:** Some of the most frequent storage pathologies associated with left ventricular hypertrophy and their chromosomal localization

Disease	MIM	Location (I)
CARDIOMYOPATHY, FAMILIAL HYPERTROPHIC, 1; CMH1	#192600	3p25.3, 1pter-p36.13, 20q11.21
GLYCOGEN STORAGE DISEASE IV; GSD4	#232500	3p12.2
CARDIOMYOPATHY, DILATED, 1S; CMD1S	#613426	14q11.2
AMYLOIDOSIS, HEREDITARY, TRANSTHYRETIN-RELATED	#105210	18q12.1
HURLER-SCHEIE SYNDROME	#607015	4p16.3
COSTELLO SYNDROME; CSTLO	#218040	11p15.5
DANON DISEASE	#300257	Xq24
MYOPATHY, X-LINKED, WITH EXCESSIVE AUTOPHAGY; MEAX	%310,440	Xq28
FABRY DISEASE	#301500	Xq22.1

When should the cardiologist suspect the presence of AFD? Cardiologists may come across such patients in outpatient visits, during clinical and instrumental evaluation in individuals with non-specific symptoms such as chest pain and arrhythmias, or after instrumental evidence of LVCH/HCM. Generally, once the presence of idiopathic LVCH is confirmed by echocardiography, cardiologists may be orientated towards a classic sarcomeric HCM. However, when investigating family and personal history, asking about extra-cardiac signs and symptoms, and after careful analysis of electrocardiographic traces, cardiologists may be lead to think about non-sarcomeric HCM. Differential diagnosis may be complicated further by the presence of the “cardiac type” of AFD in which only the cardiac phenotype is present, without any other phenotypic traits (corneal opacity, angiocheratoma, hypohydrosis, albuminuria, acroparesthesia), and in presence of a normal renal function [4]. As the disease develops lysosomes are engulfed into metabolite globotriaosylceramide (Gb3) as a result of the absence or partial activity of the a-Gal enzyme. This results in heart conduction disorders (shortening P-wave duration, atrial fibrillation and ventricular tachycardia), cardiac hypertrophy (secondary to lysosomes enlargement) causing LV mild-to-moderate concentric hypertrophy. Moreover, endothelial dysfunction leads to increased risk of myocardial infarction. Cardiac imaging highlights the mechanisms of secondary hypertrophy [5–7]. In addition to lysosomal enlargements, cardiomyocites increase their contractile elements. This hypothesis is supported by the fact that the Gb3 amount does not exceed 3% of the total myocardial mass, as shown by pathological evidence. The extension of hypertrophy of cardiomyocytes and the accumulation of Gb3, within the vacuums, correlates with the extension of the wall thickness of the left ventricle, as shown by gadolinium-based magnetic resonance [8]. Heart valves are involved in the disease. Typical thickening of the aortic and mitral valve flaps may be significantly present in both children (20%) and adults: 57% of AFD patients show impaired mitral valve and 47% show alteration of the aortic valve, with milder mitral prolapse. The aim of this paper is to provide deeper insights into the imaging data in AFD patients, irrespective of their genetic background.

### Diagnostic work-up

#### Electrocardiography

At the baseline ECG, shorter PR intervals are often the first (and sometimes the only) signs of cardiac involvement due to reduction of the P-wave duration. Specific features include:

A) Left ventricular hypertrophy. In adult patients, the electrocardiographic signs of LVH are present in more than 61% of males and 18% of females and are associated with repolarization abnormalities. The QRS duration is reported to be around 160–200 msec and the amplitude/duration product, in the 12-lead ECG, is correlated with the left ventricular mass, as measured both by MRI and pathological findings (*post-mortem*).
B) PR interval. The involvement of conduction tissue, due to the progressive infiltration by Gb3, leads to the onset of arrhythmias, showing a characteristic electrocardiographic framework. Depending on age and progression of the disease, PR is shorter in affected males (up to 11%) [3, 9]. With progression of the disease, conduction time may increase and the ECG may show a ventricular atrium block (VAB).

#### Cardiac imaging

**Fig. 1:**
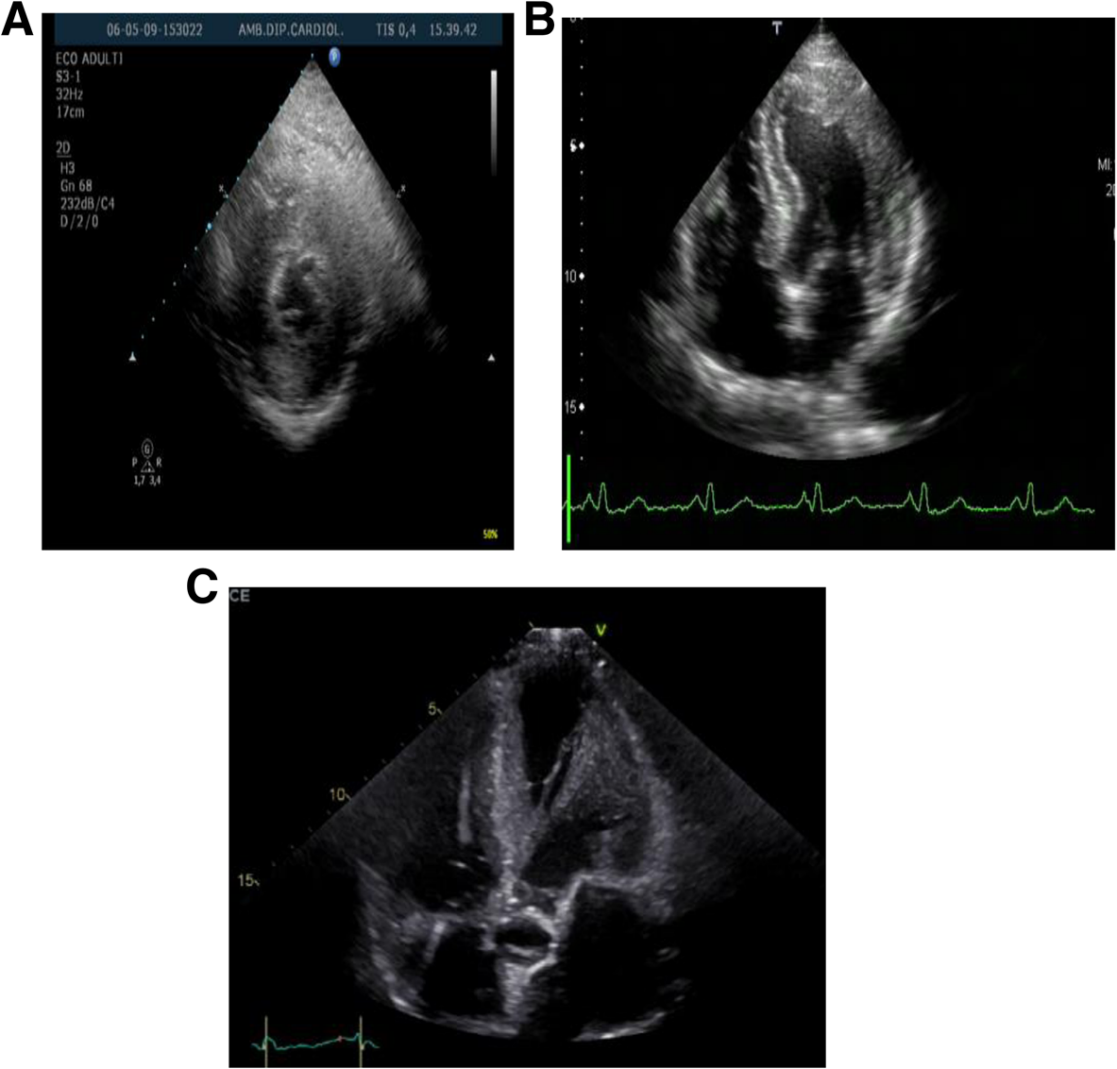
**a, b**, **c** Video. Short left echocardiogram (left) and 4-chamber apical (right) highlights the presence of concentric ventricular hypertrophy

**Fig. 2:**
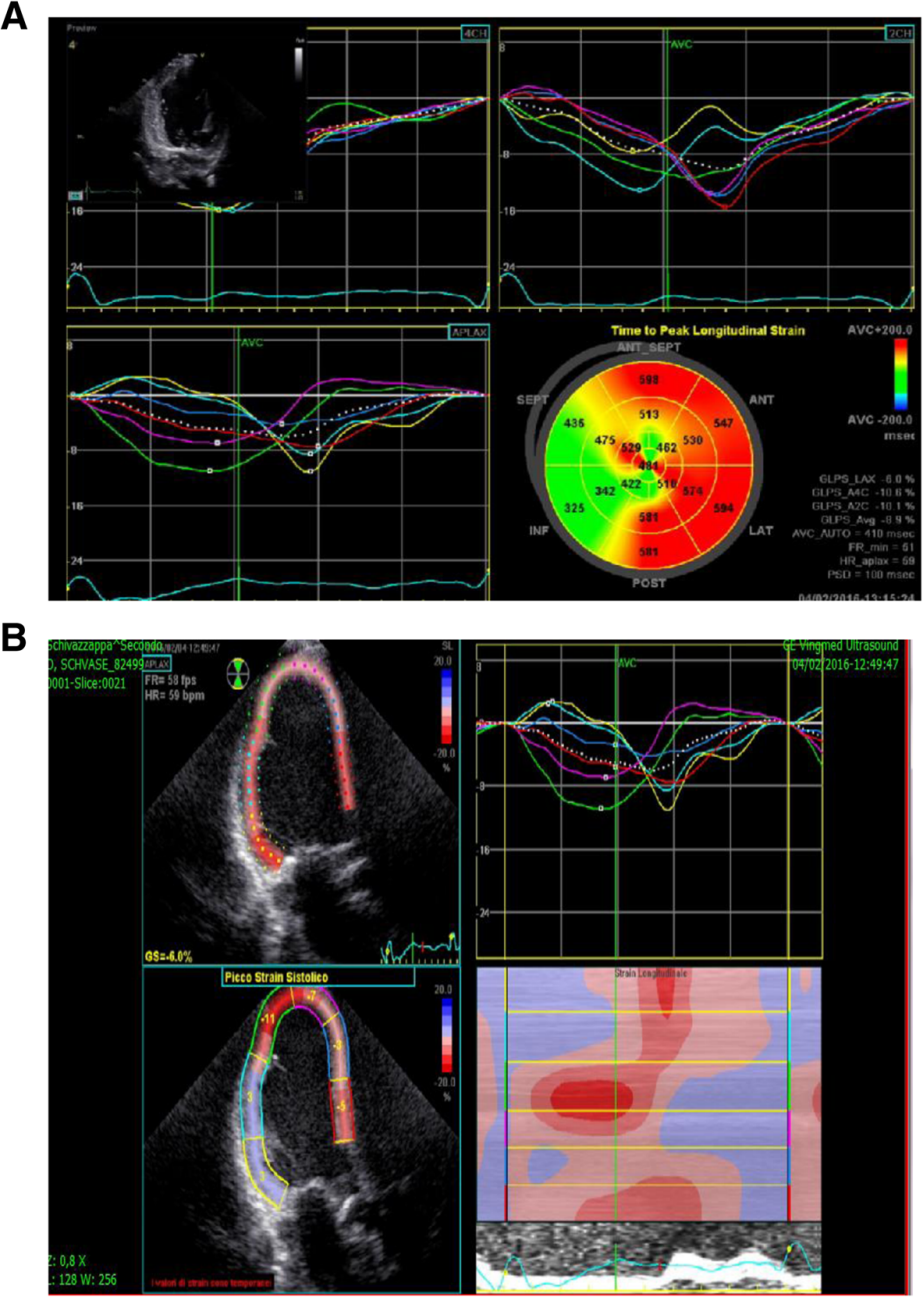
**a-b** Left Ventricle with low longitudinal strain deformation

**Fig. 3:**
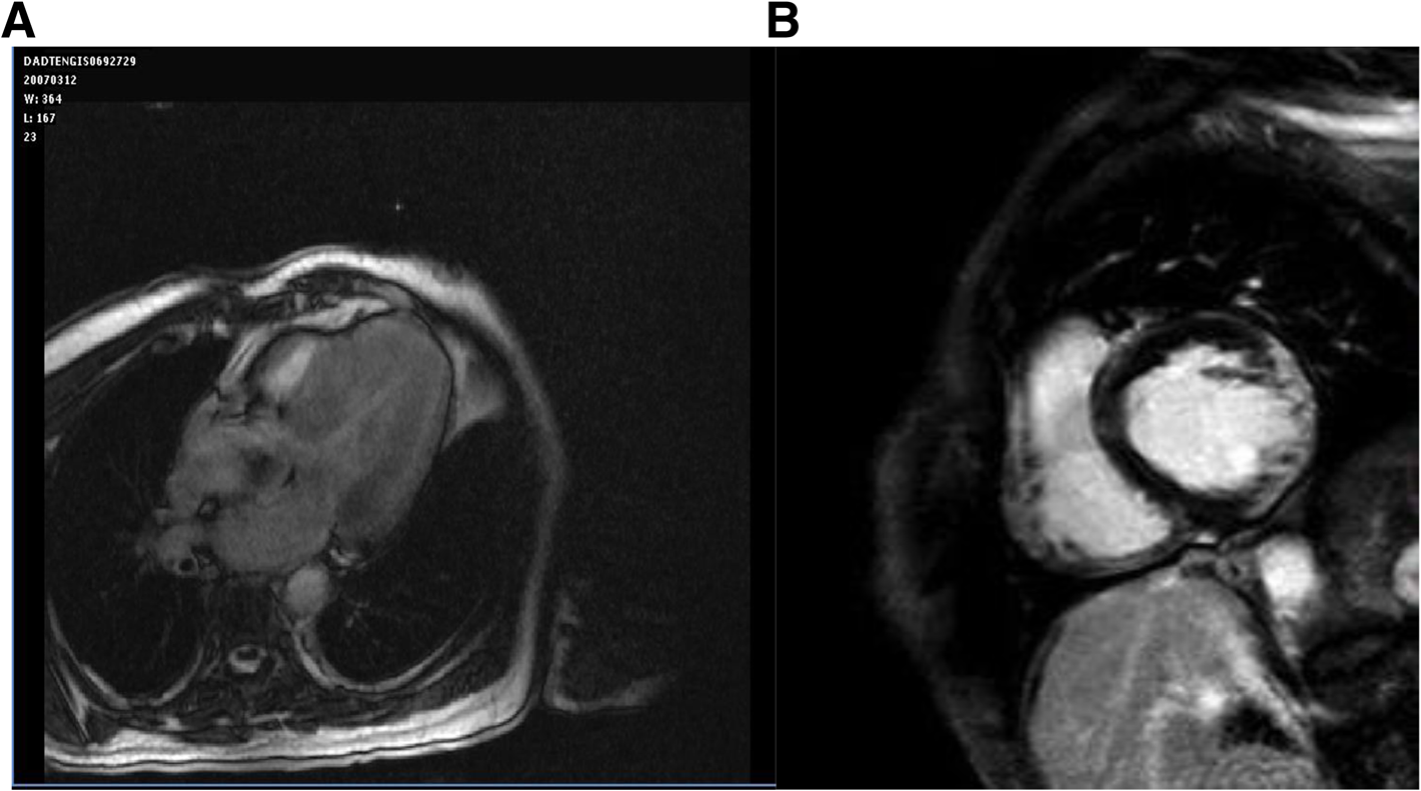
**a, b** Cardiac Magnetic Resonance shows a typical hypertrophic cardiomyopathy with hyper-enhancement relief at the left ventricular wall and enhancement relief at the left ventricular wall

A) Echocardiography
  LV hypertrophy is the morphological characteristic of the disease, often accompanied by myocardial fibrosis. A concentric hypertrophy usually occurs in adulthood of AFD patients with fibrosis of the left-lateral wall of the LV. However, eccentric hypertrophy can be detected too, as a result of the thinning of the left ventricular back-lateral basal segment, due to prevalence of the fibrous substitution. A ‘binary’ aspect of the endocardium - first detected and described by Pieroni et al. [2], may evoke the presence of AFD in subjects with LVH. Figure 1a, b, c and Video. Thickening of the papillary muscles, with or without hypertrophy of the left ventricular walls, is also prominent in these patients so that the ratio between papillary muscle size to LV circumference has been proposed [3] as an echocardiographic marker for diagnosis of AFD. Left ventricular ejection fraction (EF) is usually normal and when reduced, mortality increases. Cardiomyopathy in AFD patients has often been labeled in association with restrictive diastolic dysfunction but a restrictive pattern has proved to be extremely rare. In general, ventricular relaxation phase abnormalities are common, whereas a pseudo-normalization of trans-mitral doppler is observed in more advanced stages. Tissue Doppler Imaging (TDI) with strain deformation analysis, Zamorano et al. [10, 11] showed that velocities of the septum and lateral segment of the mitral anulus were inversely proportional to the left ventricular mass in a cohort of AFD patients. Fig. 2a,b In our series, strain rate imaging has been proven to differentiate sarcomeric HCM from AF cardiomyopathy or even cardiac amyloidosis . [12] Shank et al. [13] showed that in 16 patients, AFD had reduced longitudinal systolic strain, while there were no differences in circumferential systolic strain. Diastolic function assessment showed reduced longitudinal early diastolic SR, SR_IVR_, and E/SR_IVR (_SR(IVR) (*P* < .001) and E/SR(IVR) (*P* = .025) remained significantly different between patients with FD and controls, with sensitivity of 94% and specificity of 92% for SR(IVR) of 0.235 s(− 1) (area under the receiver operating characteristic curve, 0.953), while radial and circumferential diastolic function are not affected. Right ventricle involvement is largely parallel to the left ventricle, although it is interesting to note that the right ventricle develops fibroblast hypertrophy, as evaluated with cardiac magnetic resonance imaging [14]. Systolic excursion of tricuspid annulus (TAPSE) is the standard parameter for monitoring the function of the right ventricle and this often appears to occur normally, even in advanced stages of the disease. Valve abnormalities are frequently observed, but alterations are usually mild, although Gb3 accumulation is present in valves.
B) Cardiovascular magnetic resonance or magnetic resonance imaging (CMR/MRI).
  CMR is helpful in diagnosing HCM by identifying areas of hypertrophy, not visualized by echocardiography, providing a differential diagnosis of HCM from other causes of LVH [15]. Infiltrative cardiomyopathies, including glycogen/lysosmal storage disease, can mimic HCM as they can produce increased wall thickness. Differentiation of these ‘phenocopies’ is critical, as treatment strategies and prognosis differ compared with HCM. [16] . T1 mapping, a novel CMR imaging technique, has a great deal of power by providing further differentiation of HCM from these infiltrative cardiomyopathies. Fig. 3a,b. [17]. T1 mapping of the LV was performed in a basal and mid-ventricular short axis slice using the SAturation-recovery single-SHot Acquisition (SASHA) SSFP pulse sequence. [18] This tool has proved to be powerful in the diagnosis of cardiac involvement in AFD and also in providing insight into the biology of the pathology itself. T1 mapping is a more recent advanced tissue characterization technique whereby a low T1 represents sphingolipid deposition. A low T1 occurs in Anderson-Fabry cardiomyopathy associated with an increase of the extracellular volume (ECV), but not in amiloid infiltrative cardiomyopathy (AL,TTN) [19, 20]. In our series, CMR has proved to be the gold standard at measuring cardiac mass and function, but it’s strength is in tissue characterization. Late gadolinium enhancement in the classic location of the basal inferolateral wall is identified in up to 50% of AFD patients. It occurs in males with LVH, and females with or without LVH. It represents fibrosis in an advanced stage of the disease. Recent studies suggest that it also represents inflammation using T2 mapping and biomarkers [20]. CMR provides important supplemental information on tissue characterization using gadolinium, and provides differential diagnosis aspects among the various hypertrophic cardiomyopathies. In particular, a relaxation time (T2) is typical in patients with hypertrophic sarcomeric cardiomyopathy and hyper-enhancement relief at the left ventricular wall of the left ventricle. The extension of hypertrophy of cardiomyocytes and the accumulation of Gb3 within vacuums correlates with the extension of the wall thickness of the left ventricle, visible on gadolinium-based magnetic resonance [19].

## Conclusion

Anderson-Fabry disease, a rare X-linked disorder, is a systemic lysosomal disorder. AFD cardiomyopathy can mimic hypertrophic sarcomeric cardiomyopathy (HCM) or amyloid cardiomyopathy. Differential diagnosis, by multidisciplinary approach and by incorporating specific cardiac signs, has important therapeutic and prognostic implications because AFD patients have benefited from an enzyme replacement therapy (ERT) since 2001. Cardiac imaging plays an important role in detecting this peculiar disease in patients with cardiac septum/wall thickening, both in the classic AFD and in its cardiomyopathy variant (AFD cardiomyopathy) form, therefore, thanks to timely diagnosis, avoiding end-stage renal failure and heart failure.

## Abbreviations

AFD: Anderson-Fabry Disease
AVB: Atrio-Ventricular Block
EF: Left ventricular Ejection Fraction
ERT: Enzyme Replacement Therapy
LVCH: Left Ventricle Cardiac Hypertrophy
LVM: Left Ventricular Mass
PM: Pace-maker
TAPSE: Systolic excursion of tricuspid annulus
TDI: Tissue Doppler Imaging
